# Low concentrations of local honey modulate Exotoxin A expression, and quorum sensing related virulence in drug-resistant *Pseudomonas aeruginosa* recovered from infected burn wounds 

**DOI:** 10.22038/ijbms.2019.33077.7902

**Published:** 2019-05

**Authors:** Akhter A Ahmed, Fraidoon A Salih

**Affiliations:** 1 Department of Biology, College of Science, Salahaddin University-Erbil, Erbil, Iraq

**Keywords:** Biofilm, Exotoxins, Honey, P. aeruginosa, Quorum-sensing, Virulence

## Abstract

**Objective(s):**

Honey’s ability to kill microorganisms and even eradication of chronic infections with drug-resistant pathogens has been documented by numerous studies. The present study is focused on the action of honey in its sub-inhibitory levels to impact on the pathogens coordinated behaviors rather than killing them.

**Materials and Methods:**

The impact of local honey on the quorum sensing related virulence of multidrug resistant *Pseudomonas aeruginosa* burn isolates was investigated by detection its effect on the virulence, biofilm formation and expression of quorum sensing related and exotoxin A genes.

**Results:**

Experiments to characterise and quantify the impact of honey on the *P. aeruginosa* quorum sensing networks showed that the expression of exotoxin A ( *ETA*), las and rhl glucons reduced by low concentrations of honey including the associated virulence factors.

**Conclusion:**

Our results indicated that honey fights infections either by its bactericidal components which vigorously kill cells or by weakening bacterial coordination and virulence through interruption of quorum sensing system.

## Introduction

As the 21^st^ century commences, excessive and indistinctive use of antibiotics has led to the emanation of multi-drug resistant (MDR) bacterial strains (1). Today, the universal concern is of diminished capability to fight microbes while we are entering a post-antibiotic epoch. 

The fact that 16 million people die every year due to the infectious diseases caused by MDR strains led to the need for alternative strategies to combat them (2, 3). One strategy to treat antibiotic-resistant strains which is less likely to assess a selection pressure for development of resistance is by the development of new mechanisms of antipathogenic treatments that act to weaken the virulence expression (4, 5). 

The promising approach to improve unique antipathogenic treatment is by blocking the communication between cells which is generally identified as quorum sensing (QS). The QS is a mode of bacterial signalling that depends on the production of small diffusible molecules known as “autoinducers” during the bacterial growth phase. Once a threshold concentration is reached, these auto-inducers will bind to their transcriptional regulators and form a complex that regulates the expression of many genes particularly those control virulence (6). N-acyl homoserine lactone (AHL) and small post-transnationally processed peptides are among the most investigated intra-species auto-inducers in Gram-negative and Gram-positive bacteria respectively (3, 4, 7).

*Pseudomonas aeruginosa *is an ordinarily adaptive pathogen that causes persistent infections in the burn, genetic disease cystic fibrosis and immune-compromised patients (8).

Las and Rhl are the two well-studied *P. aeruginosa *QS systems, N-butanoyl homoserine lactone and -3-oxo-dodecanoyl homoserine lactone function as autoinducers (AI), and *LasR* and *RhlR* act as transcriptional regulators. (9). Many virulence factors of* P. aeruginosa* are under the control of QS systems like secretion of alkaline and Las A protease, LasB elastase, pyocyanin, exotoxin A and biofilm formation (10). 

Significant efforts have been made to ascertain compounds with inhibitory activity of QS systems. The first described anti-QS agent which isolated from the red algae (*Delisea pulchra*) was halogenated furanones. Awkwardly, these are excessively reactive and may be very toxic to be used for human. Accordingly, there is a crucial necessity for the characterization of new non-toxic anti-QS inhibitors (11).

It is becoming well known that honey impacts on the virulence of bacterial pathogens in addition to influencing cellular structure and metabolism (12).

Although several studies afford promising evidence for the effectiveness of honey in treating pseudomonal infections, there is a need to explore further this issue, mainly related to the anti-QS and anti-biofilm properties of honey. As many virulences of *P. aeruginosa* have been investigated but there are no studies concerning the effect of honey on the exotoxin A at the level of expression and to investigate the role of local honey (LH) in attenuating virulence factors through reducing the expression of networks of *Las *and *Rhl *genes in *P. aeruginosa* this study was conducted.

## Materials and Methods

***Honey***

Raw and unprocessed samples of honey were obtained from the mountains of Kurdistan, Iraq and cold processed at temperatures below 30 ^°^C. The honey sample was placed in glass containers and kept in the dark at room temperature until use.

***Specimens collection and samples sources***

A total of 25 non duplicate isolates of *P. aeruginosa *were attained from wounds swabs submitted to be tested for bacteriology from hospitalised patients with burn admitted to the West Emergency Hospital Erbil City, Iraqi Kurdistan. With a sterile cotton swab, the specimens were taken from ulcers and exudates of the burn. The swabs were primarily inoculated onto Cetrimide and MacConkey agar medium (acumedia, Neogen, USA) and incubated at 37 ^°^C for 24 hr. From the individual colonies, *P. aeruginosa* was identified by various conventional diagnostic and biochemical tests as described previously (13). Bacterial isolates further identified by Vitek II automated system (bioMérieux Marcy l’´Etoile, France) (Vitek Systems Version: 06.01) with the ID-GNB card for identification of Gram-negative bacilli. Furthermore, *P. aeruginosa* isolates were tested for their susceptibility to a panel of antimicrobials (Amikacin, Ceftazidime, Chloramphenicol, Ciprofloxacin, Doxycycline, Meropenem, Netilmicin and Tobramycin) by Vitek II automated system and disc diffusion method, then the most resistant isolate was chosen for all experiments throughout the study. The identified colonies were then inoculated into sterile tubes containing 1 ml of sterile Tryptic Soy Broth (TSB) (Oxoid) containing 30% glycerol and stored at -70 ^°^C for further study.

***Minimum inhibitory and bactericidal concentrations (MIC and MBC)***

 Broth microdilution method was used to determine the MIC for local honey against the identified MDR *P. aeruginosa* isolates (14). Ten µl of *P. aeruginosa* cells in stationary-phase equilibrated to OD_550_=0.5 inoculated to 100 µl Nutrient broth (NB; Oxoid) containing different concentrations (1–20 % v/v, in increments of 2%) of local honey in the wells of a polystyrene microtitre plate (MTP). The MTPs were incubated aerobically at 37 ^°^C for 24 hr. The lowest concentration with no evident growth was determined as MIC. To establish the MBC, from the wells with no visible growth 100 µl was streaked on Nutrient agar (NA; Oxoid) plates and incubated aerobically at 37 ^°^C for 24 hr. The concentration at which no growth was detected on NA plates was determined as MBC. Subinhibitory concentrations (SICs) were determined as the level below the MICs and further used to assess the anti-biofilm and anti-virulence activity in the isolated* P. aeruginosa* strains. Three biological replicates were considered on distinct occasions. 

***Growth and viability***

*Bacterial growth curve analysis*

To emphasize the anti-QS potency and to confirm the non-growth-inhibitory action of the local honey flask incubation assay was performed (15). One percentage overnight culture of MDR isolate of* P*. *aeruginosa *(OD adjusted to 0.5 at 550 nm) was transferred to 50 ml of Luria-Bertani (LB) broth supplemented with a SIC of honey in a 100 ml Erlenmeyer flasks. The flasks were incubated at 37 ^°^C in a rotatory shaker with 180 rpm agitation for 24 hr. OD_550_ was observed at hourly interims for up to 24 hr. Uninoculated control flasks were used as a blank for the concentration of honey, and the change in optical density was considered over time.

The impact of local honey on the *P. aeruginosa* viability was also observed by determining population numbers by total viable counts (TVCs) as described by Roberts *et al.* (14). Stationary-phase of *P. aeruginosa* cells (5x10^6^ cells per ml) were transferred to 100 ml Erlenmeyer flasks containing 20 ml NB with a SIC of honey. The flasks were incubated for eight hours at 37 ^°^C with 150 rpm agitation in a rotary shaker. At hour intervals, samples were diluted by 0.25% Ringer’s solution (Oxoid), inoculated on NA plates, and the plates were incubated at 37 ^°^C for 24 hr. The total number of surviving bacteria was determined. Three biological replicates were considered on separate occasions, and the standard error was calculated.

***Protease assay***

Protease activity was determined by inoculating honey treated, and untreated *P. aeruginosa* separately on LB solid medium containing 2% skim milk. After incubation at 37 ^°^C up to 48 hr, a clear zone surrounding the growth area indicates casein proteolysis (16). 

***Azocasein degrading proteolytic activity***

Proteolytic activity of cell-free supernatant of *P. aeruginosa* was determined by azocasein assay as described by Kessler *et al.* (17). Briefly, 150 μl *P. aeruginosa* culture supernatants of treated and untreated with the SIC of honey were added to 1 ml of 0.3% azocasein (Sigma, USA) in 0.05 M TrisHC1 and 0.5 mM CaCl_2_ (pH 7.5), and incubated at 37 ^°^C for 15 min. To stop the reaction trichloroacetic acid (TCA l0%, 0.5 ml) was added, centrifuged, and the absorbance was measured at 400 nm.

***Pyocyanin assay***

Briefly, overnight cultures of an OD_550_ of 0.5 in LB medium was diluted 1:10 in pyocyanin production broth (PPB; 2 % peptone 1 % K_2_SO_4_, 0.3 % MgCl_2_). Twenty millilitres of the diluted culture with a SIC of local honey was cultivated in PPB for 24 hr. Pyocyanin was extracted by 3 ml chloroform; the blue colour layer was re-extracted into one ml 0.2 M HCl, yielding a red layer on the top. The absorbance was measured at 520 nm, and the concentration of pyocyanin in the absence and presence of honey was determined by multiplying the absorbance by 17.07 (18).

***Swarming and swimming motility assay***

Swarming and swimming assays were measured as described previously by Deziel *et al.* (19). Briefly, swarming media plates consisting of 1% peptone, 0.5% NaCl, 0.5% agar and 0.5% of filter-sterilised D-glucose with SIC of honey were point inoculated at the centre with overnight cultures of the bacterial isolates, a plate without honey was maintained as a control. The inoculated plates were incubated at 30 ^°^C for 24 hr and to detect the extent of swarms the diameter of the motility swarms was measured. For swimming assay, the centre of the swimming media plates containing 1% tryptone, 0.5% NaCl and 0.3% agar supplemented with a SIC of honey was point inoculated with the overnight cultures of the bacterial isolates and incubated at 30 ^°^C for 24 hr. The swimming migration was measured by following swim zones of the bacterial cells after, swim agar plate without the addition of honey was maintained as a control.

***Assay for biofilm inhibition***

The impact of honey on biofilm formation was detected by the microtiter plate assay (20). Briefly, overnight cultures of *P. aeruginosa* re-suspended in fresh LB broth in flat bottoms polystyrene microtiter plates (Costar/USA) in the presence and the absence of SIC of honey and incubated in a static condition at 37 ^°^C for 24 hr. The liquid cultures in the well plates were removed, by phosphate buffer saline (PBS) the wells were washed three times and stained with 1% crystal violet solution. The wells then washed with distilled water and quantified by solubilising the stain in ethyl alcohol. The adhesion ability to the abiotic surface was determined by reading the coloured suspension density by an Elisa reader (Epson, Biotek, UK) at a wavelength of 490 nm. 

***In situ visualisation of biofilms***

The biofilms visualised by using the method mentioned by Al-Shabib *et al.* (21). One per cent of overnight cultures of the test isolates adjusted to 0.4 OD at 600 nm was added to 1 ml of fresh LB broth containing 1 cm^2^ cover glasses with honey at SICs, and untreated cultures serve as control. After incubation, the coverslips were dipped thrice with distilled water, stained with 1% crystal violet solution, dried and were placed on slides with the biofilm pointing up, and the biofilms were visualised at magnifications of 40X of the light microscope.

***RNA extraction***

To evaluate the expression of the *ETA*, *las* and *Rhl* genes, RNA was extracted from bacterial cells in mid-exponential growth phase grown in the presence and absence of SIC of honey according to the extraction protocol described by the manufacturer (Total RNA Purification Kit, Jena Bioscience-Germany). RNase-free DNase I (Promega, USA) was used to remove the residual genomic DNA from the RNA fraction. The concentration and the purity of the total RNA were measured by ultraviolet absorption (260/280 nm) using IMPLEN Nanodrop spectrophotometer.

***Real-time reverse transcription polymerase chain reaction (RT-PCR)***

Relative expression was measured by one-step quantitative RT-PCR SuPrimeScript RT-PCR Kit with SYBER Green l (Genaid Bio) following manufacturer’s conditions in a 20 μl total reaction volume. RT-PCR was performed using the PCR^max ^Eco 48 Real-Time PCR system. The reaction procedure was performed as follows: 50 ^°^C for 20 min (cDNA synthesis), 95 ^°^C for 10 min (initial denaturation), 40 cycles of 95 ^°^C for 15 sec (denaturation), and 60 ^°^C for 60 sec (annealing /extension). RT-PCR amplifications were performed in triplicate. Primer sequences for *P. aeruginosa* quorum sensing and *ETA* genes were used as depicted previously (Table 1). Comparative critical threshold (Ct) real-time PCR was used to calculate relative gene expression, the range of applicant gene expression between honey treated and untreated samples was compared to study relative gene expression, and the effect of local honey treatments on each gene.

***Statistical analysis***

The paired t-test for significance was done to compare between control strains and the treated clinical isolates.

## Results

***Inhibition of planktonic P. aeruginosa by LH and growth analysis***

The MIC of honey was determined to select the SIC to investigate its effect on the growth and quorum sensing regulated functions.

**Figure 1:**
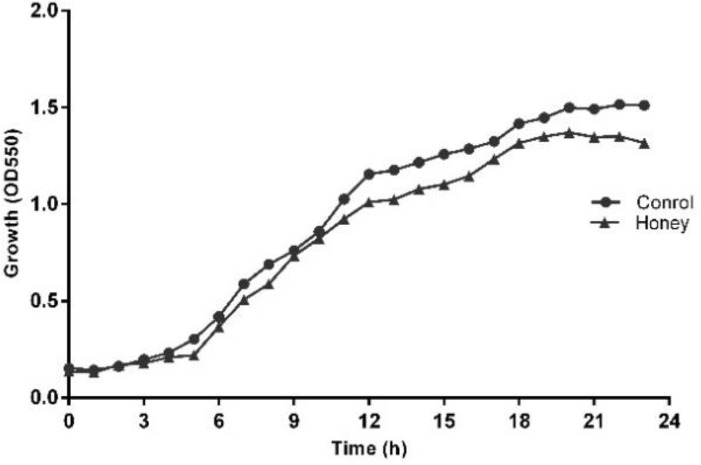
Growth curves of *Pseudomonas aeruginosa* isolate in the presence of LH over 24 hr; points denote means from three biological replicas

**Figure 2:**
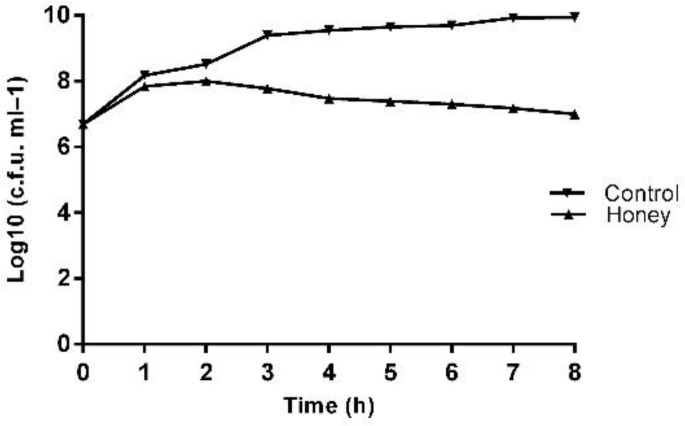
TVCs of *Pseudomonas aeruginosa* isolates treated with LH over 8 hr; points denote the mean from three biological replicas

The MIC and MBC of honey against *P. aeruginosa *clinical isolate (which was resistant all antimicrobials tested in the current study except Amikacin and Ciprofloxacin) were 1% (v/v) and 3% (v/v), respectively. Growth curves with SIC resulted in no significant reduction in the growth rate and whole cell number (Figure 1) compared to untreated cells. TVCs data with SIC 1% (v/v) of local honey revealed a slight drop in the total bacterial number (Figure 2) over an eight hours treatment period at each time point when compared to untreated values.

**Figure 3:**
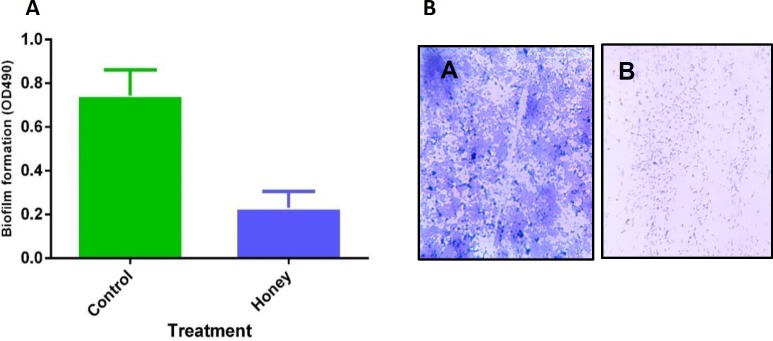
(A) Quantitative measurement of *Pseudomonas aeruginosa *biofilm reduction by measuring absorbance at 490 nm. Data are presented as mean±SE. (B) Light microscopic images of biofilms stained with crystal violet in the absence (A) and presence (B) of SIC of local honey

**Figure 4:**
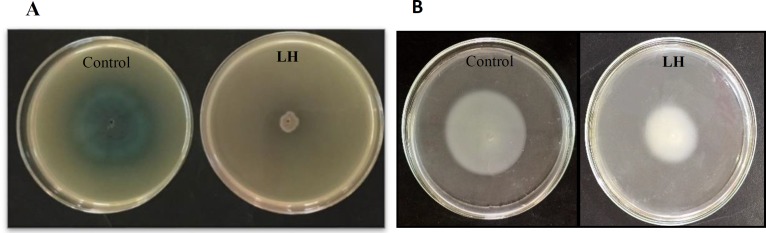
Honey impacts the production of QS-related virulence factors in *Pseudomonas aeruginosa*. Local honey (LH) decreases swarming (A) and swimming (B) motility when compared with untreated control

**Figure 5:**
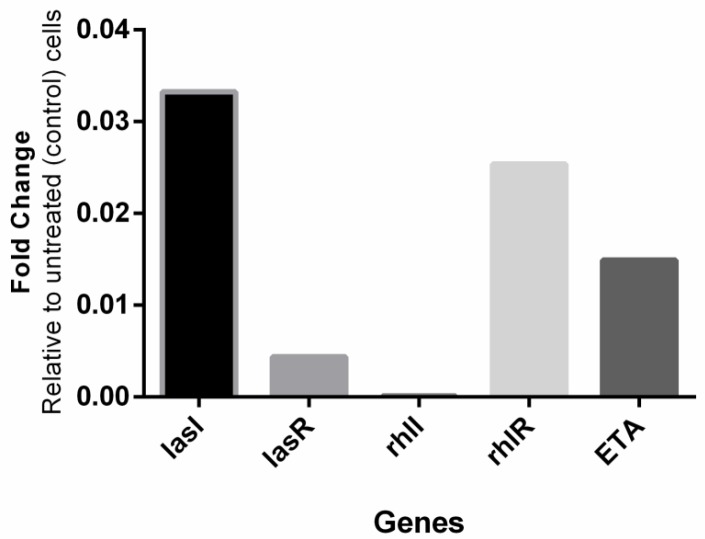
Transcriptional profiles of quorum sensing and *ETA* gene expression of clinical isolates of *Pseudomonas aeruginosa* when treated with SIC of honey. Transcriptional profiles were measured by RT-PCR

**Table 1:** Primer sequences used in current study

**Target** **Gene**	**Primer sequence**s (**5' to 3')**	**Reference(s) of source**
*Las I*	Fw: ATGATCGTACAAATTGGTCGGC Rv: GTCATGAAACCGCCAGTCG	(22)
*Las R*	Fw: ATGGCCTTGGTTGACGGTT Rv: CAAGATCAGAGAGTAATAAGACCCA	(23)
*RhlI*	Fw: CTTGGTCATGATCGAATTGCTC Rv: ACGGCTGACGACCTCACAC	(22)
*RhlR*	Fw: CAATGAGGAATGACGGAGGC Rv: GCTTCAGATGAGGCCCAGC	(22)
*ETA*	Fw: GACAACGCCCTCAGCATCACCAGC Rv: CGCTGGCCCATTCGCTCCAGCGCT	(24, 25)

Fw, forward primer; Rv, reverse primer.

**Table 2:** Effect of SIC of local honey on the reduction of P. aeruginosa virulence factors

*P. aeruginosa*	aTotal protease	bExoprotease	cPyocyanin production	dMotility	
				Swarming	Swimming
Control	0.706 ± 0.014	25.33±0.88	5.317 ± 0.044	36.7 ±3.28	54.33 ±1.202
Honey Treated	0.213 ± 0.008**	0±0.0**	0.375 ± 0.015***	15.7 ± 0.333 ***	24 ±0.577**

Data denote mean values of three independent experiments,

**significance at *P*≤ 0.01,

***significance at *P* ≤ 0.001.

a Total protease was expressed as the absorbance at OD420.

b Exoprotease production was expressed as diameter in mm.

c Pyocyanin concentrations were expressed as µg of pyocyanin produced per µg of total protein.

d Swarming and swimming motility were expressed as diameter in mm**.**

***Honey affects quorum sensing regulated virulence factors***

The effect of honey on the virulence factors controlled by QS was investigated. The honey was tested for its ability to decrease or inhibit the secretion of bacterial extracellular proteases, which are notorious to be regulated by *MvfR* and the *las *operon part of the AHL network (26). When *P. aeruginosa *was cultured on plates of skim milk supplemented with SIC of honey, the clear halo around colonies almost wholly eliminated due to break down of casein. Control plates showed a 25-mm halo, whereas no halo was detected around the colonies on the honey plates (Table 2). The activity of total protease was also decreased significantly at the tested concentration. The local honey was further investigated for its reduction ability of pyocyanin excretion which is a toxic blue-green substance limited to *P. aeruginosa* (27). Table 2 shows the results for *P. aeruginosa *grown in LB broth supplemented with 1% honey and control with no honey, a significant reduction of pyocyanin production was observed in the honey-treated culture. Effect on these distinct quorum sensing regulated virulence factors proposes that honey impacts the expression of the regulator genes. This effect would designate an essential interaction between honey and the quorum sensing networks of *P. aeruginosa.*

***Reduction of clinical isolates of P. aeruginosa biofilm formation by LH without inhibition of planktonic growth***

The ability of local honey to reduce or inhibit the production of biofilm in the clinical isolate of *P. aeruginosa* was investigated in 96-well MTPs. Local honey significantly reduced biofilm formation in the studied bacteria. Notably, the local honey at 1% (v/v) inhibited biofilm formation (Figure 3A), while the honey at 3% (v/v) completely inhibited the bacterial growth (data not shown). 

The results of biofilm reduction interrelated definitely with swarming and swimming inhibition (Figure 4), as motility has an essential role in adhesion and development of biofilms. Unlike antimicrobials agents, it is desirable that potential antibiofilm or anti-virulence compounds do not inhibit bacterial growth, as this could lead to bacterial drug resistance.

***In-situ visualisation of biofilm inhibition***

The images of light microscopy revealed a thick layer of biofilm on the untreated coverslips, stained easily with crystal violet, and visualised under the light microscope. Whereas honey treated with SIC coverslips showed a diminishing of *P. aeruginosa* biofilm formation (Figure 3B). 

***Repression of quorum sensing genes by honey***

Given honey’s impact on quorum sensing regulated virulence factors, the subsequent step was to study its effect on QS genes. Quantitative reverse transcriptase real-time PCR was used to conclude whether the bacterial inability to produce autoinducers and exotoxin A following honey treatment (1 %, v/v) would target their genes. The investigated genes in this part of the study were *ETA* and quorum sensing genes *las I, lasR, rhlI*, and *rhlR* those are involved in virulence and biofilm development. The critical threshold cycles (Ct) values between biological samples were standardized against untreated cells, and relative changes in the copy number to untreated control cells were analyzed. 

Consequently, we investigated whether honey directly impacts the expression of quorum sensing and *ETA* genes. A notable reduction was indicated in the expression of the particular genes in the bacterial cells treated with local honey, while cell growth was unaffected (Figure 5). The observed reduction of QS genes and signal molecules besides the influences on downregulation virulence factors in the *las* and *rhl *operons shows a wide-range impact on QS networks.

## Discussion

The occurrence of MDR bacteria has motivated studies for weakening virulence differently through QS inhibition procedures instead of bactericidal and bacteriostatic strategies (28-30). 

*P. aeruginosa* is considered one of the most predominant colonizers of leg ulcers and burns (14). It is recognised among the most critical respiratory bacterial pathogens causing substantial morbidity and mortality (31); to combat this MDR organism unique therapeutic approaches are required. 

It was realised that honey could be utilized as an appropriate antimicrobial treatment for the infected wounds, with permitted therapeutic products becoming accessible in the 1990’s (32). Besides its essential role in traditional medication, scientists accept honey as an innovative active medicine for various types of illnesses. Among the most recognized properties of honey is the antibacterial activity (33). Honey’s impact on the bacterial pathogens is well documented, several studies reported the bactericidal and bacteriostatic activities of honey against *P. aeruginosa *(14, 34). 

New approaches are becoming increasingly apparent to honey’s impact on the virulence of bacterial pathogens rather than traditional bactericidal or bacteriostatic medications. More directly the effect on genes that control the activation of virulence is of interest investigation to combat microbes resistant to traditional treatments.

This study characterises local honey’s ability to interfere with virulence factors in addition to its interaction with the quorum sensing system of *P. aeruginosa*, using 1% (v/v) as the sub-inhibitory concentration to test its effect on surviving bacterial cells systematically. Our strategy considerably varies from using high concentrations of honey to determine the bactericidal activities and highlights a different line of knowledge concerning the honey’s remarkable ability to combat microbes. The results obtained by the current study emphasise that low concentration of local honey could reduce or inhibit the expression of many virulence factors of *P. aeruginosa* clinical isolates. It is significantly satisfying that our local honey affects MDR isolates at lower concentrations than recorded for Manuka honey (known as medical honey) (14, 34). Even though the bioactive components are reduced when honey is diluted to low concentrations, the sugar content still may reduce or inhibit the QS related genes. It was reported that sugars like glucose and fructose impact QS related virulence in bacteria as honey did (35, 36).

To establish the QS inhibitory, the effect of the honey was evaluated on quorum sensing regulated virulence factors of clinical isolates of the studied bacterium. Quorum sensing signalling in the* P. aeruginosa* is AHLs based and involves two systems which are, the las and rhl systems that control the production of virulence factors such as exotoxins, exoproteases, pyocyanin, and contribute in the formation of biofilms (37).

Proteases enzymes participate significantly in the *P. aeruginosa *pathogenesis; degrade host tissues and enhance the bacterial growth and invasiveness (38). Significant inhibition of proteases was observed in isolates treated with SICs of honey. Pyocyanin which is a secondary metabolite of *P. aeruginosa* damages the neutrophil-mediated host defence which causes severe toxic effects (39). The reduction of pyocyanin production is congruous with the remarkable decrease in the expression of the las and rhl systems; honey similarly prohibited the secretion of extracellular proteases which are controlled by las operon (19). This operon is recognized to be regulated by *mvfr *and lasR systems. Our results on the reduction of virulence factors find support from the findings of Wang *et al.* (36).

Flagella-motivated swarming movement is a quorum sensing dependent virulence function that plays an essential role during the development of biofilms in the attachment of a cell to surfaces (40). The decrease in the swarming movement is indicative of honey’s ability to inhibit flagellar synthesis. 

Bacterial biofilm is sessile microbial populations that attach to surfaces by extracellular polymeric substances. A biofilm is an organized connotation of bacteria implanted in a self-produced polymer matrix comprising of polysaccharide, protein and DNA (41). Biofilms are prevalent in natural, medical, and engineering environments (42). Pathogenic bacteria form biofilms that cause many disorders to human health, like periodontitis , cystic fibrosis and prostatitis which is due to their increased resistance to antimicrobial treatments (43).

 The cells of *P. aeruginosa* biofilm are described to be more resistant to antimicrobials than planktonic cells, which often difficult to destroy them from infected patients. The development of biofilms in* P. aeruginosa *is regulated by several factors, and one of the primary controlling mechanisms is the QS system (44). 

A remarkable decrease in the expression of lasI and rhlI quorum sensing paths in* P. aeruginosa *was indicated. Both systems are part of the *lasR *and *rhlR *systems that structure the AHL network (27). The rhlR system is linked *mvfr* and regulates an additional array of virulence factors such as the secretion of proteases, movement, and biofilm development (45, 46). 

This study demonstrated that a concentration of local honey at 1% could decrease the expression of virulence and quorum sensing genes and biofilm formation in *P. aeruginosa* without affecting its growth. This mechanism differed from a previous theory which suggested that bacterial growth inhibition reduced biofilms by antimicrobial components, such as methylglyoxal, hydrogen peroxide, or bee peptide defensin-1 (47, 48). 

Some virulence factors of *P. aeruginosa *enable it in adhering to tissue surfaces, damaging tissue for nutrition supply and dissemination and increasing its survival rate (49). One of them is exotoxin A, which belongs to the family mono-ADP-ribosyl transferase and has enzymatic activity (50) and it is produced by most of *P. aeruginosa* clinical strains (51). This study investigated the ability of honey to reduce the expression of exotoxin A for the first time. A notable reduction in the fold change is observed. Moreover, it is essential for the toxin to be in an adequate concentration in the surroundings for efficient killing (52). 

 The ability of various kinds of honey to fight microbes might be due to at least two corresponding mechanisms. The first mechanism related to the destruction of organisms by direct biocidal factors. The other mechanism is referred to honey’s anti-virulence activity, by inhibition the expression of genes allied with stress tolerance, production of virulence, and multicellular behaviours of the organism such as quorum sensing and biofilm development (36). The latter mechanism will ultimately decline bacterial organization, reduce their existence abilities, and restrict virulence mechanisms. The present study focused on the anti-virulence and antibacterial activities, depending on the review, this is the first attempt to evaluate the effects of Kurdish honey on the *P. aeruginosa* at both inhibitory activity and molecular levels. Additional experiments are needed to analyse the honey to determine components that distinguish its contributing factors and independent components.

However, if both the las and the rhl cell-to-cell signalling systems are congested, *P. aeruginosa* might be unable to re-establish the production of cell-to-cell signalling dependent virulence factors; this approach may efficiently reduce virulence factors production and high death rates associated with *P*. *aeruginosa.*

## Conclusion

Direct inhibitory effects of the kurish honey against* P. aeruginosa* were indicated in the current study which was further sustained by structural and morphological investigations. The findings of gene expression in response to honey’s treatment revealed downregulation of many genes contributed in quorum sensing and exotoxin A in *P. aeruginosa*. Honey’s ability to downregulate the expression of studied genes is valuable in it’s therapeutic and prophylactic use. To the best of our information, these results afford the first report that local honey is effective against* P. aeruginosa* with double mechanisms that include the direct inhibition of growth and the downregulation of some of the virulence-related genes. 
